# Asthma and COPD in cystic fibrosis intron-8 5T carriers. A population-based study

**DOI:** 10.1186/1465-9921-6-113

**Published:** 2005-10-09

**Authors:** Morten Dahl, Anne Tybjærg-Hansen, Peter Lange, Børge G Nordestgaard

**Affiliations:** 1 Department of Clinical Biochemistry, Herlev University Hospital, DK-2730 Herlev, Denmark; 2 Department of Clinical Biochemistry, Rigshospitalet, Copenhagen University Hospital, DK-2100 Copenhagen Ø, Denmark; 3 Department of Respiratory Medicine, Hvidovre University Hospital, DK-2650, Hvidovre, Denmark; 4 The Copenhagen City Heart Study, Bispebjerg University Hospital, DK-2200 Copenhagen N, Denmark

## Abstract

**Background:**

Carriers of cystic fibrosis intron-8 5T alleles with high exon-9 skipping could have increased annual lung function decline and increased risk for asthma or chronic obstructive pulmonary disease (COPD).

**Methods:**

We genotyped 9131 individuals from the adult Danish population for cystic fibrosis 5T, 7T, 9T, and F508del alleles, and examined associations between 11 different genotype combinations, and annual FEV_1 _decline and risk of asthma or COPD.

**Results:**

5T heterozygotes vs. 7T homozygous controls had no increase in annual FEV_1 _decline, self-reported asthma, spirometry-defined COPD, or incidence of hospitalization from asthma or COPD. In 5T/7T heterozygotes vs. 7T homozygous controls we had 90% power to detect an increase in FEV_1 _decline of 8 ml, an odds ratio for self-reported asthma and spirometry-defined COPD of 1.9 and 1.7, and a hazard ratio for asthma and COPD hospitalization of 1.8 and 1.6, respectively. Both 5T homozygotes identified in the study showed evidence of asthma, while none of four 5T/F508del compound heterozygotes had severe pulmonary disease. 7T/9T individuals had annual decline in FEV_1 _of 19 ml compared with 21 ml in 7T homozygous controls (t-test:P = 0.03). 6.7% of 7T homozygotes without an F508del allele in the *cystic fibrosis transmembrane conductance regulator *gene reported asthma vs. 11% of 7T/9T individuals with an F508del allele (χ^2^:P = 0.01) and 40% of 7T homozygotes with an F508del allele (P = 0.04). 7T homozygotes with vs. without an F508del allele also had higher incidence of asthma hospitalization (log-rank:P = 0.003); unadjusted and adjusted equivalent hazard ratios for asthma hospitalization were 11 (95%CI:1.5–78) and 6.3 (0.84–47) in 7T homozygotes with vs. without an F508del allele.

**Conclusion:**

Polythymidine 5T heterozygosity is not associated with pulmonary dysfunction or disease in the adult Caucasian population. Furthermore, our results support that F508del heterozygosity is associated with increased asthma risk independently of the 5T allele.

## Background

Asthma and chronic obstructive pulmonary disease (COPD) are caused by complex interactions between environmental and genetic factors. A putative genetic risk factor for asthma and COPD is the *cystic fibrosis transmembrane conductance regulator (CFTR) *gene [1-3]. This gene encodes a cAMP-regulated channel with chloride activity in pulmonary epithelia. When channel activities are absent, cystic fibrosis with life-threatening airways obstruction due to thickened secretions and secondary pulmonary infection develop [4]. The most common cause of cystic fibrosis is homozygosity for the phenylalanine-508 deletion (F508del), explaining about 70% of cystic fibrosis worldwide [4,5].

We previously showed that persons heterozygous for a F508del deletion are overrepresented among people with asthma [1,6]. Another more common variant, the 5T allele, could likewise be involved in asthma [7] or COPD. This variation is in the polythymidine tract of the *CFTR *gene and has mainly been associated with congenital bilateral absence of the vas deferens, a monosymptomatic form of cystic fibrosis [8-10]. However, it may also be associated with increased risk of obstructive lung disease, particularly bronchiectasis [9-14]. Because most previous studies on lung disease in 5T carriers were based on case patients [2,9-24], currently we know little about the risk for obstructive lung disease in 5T carriers in the general population.

Three common alleles are known in the polythymidine tract, 5T, 7T, and 9T. The polythymidine tract is situated in intron-8 near the acceptor splice site for exon-9 [25,26]. The shorter this polythymidine tract is, the more often exon-9 is skipped from CFTR mRNA. Transcripts missing exon-9 increases from 1%–13% in 9T homozygotes [27-29] to 12%–25% in 7T homozygotes [13,27-30] to 66%–90% in 5T homozygotes [13,27,31,32]. CFTR mRNA without exon-9 leads to a protein with no chloride channel activity [33,34]. Thus, carriers of 5T with high exon-9 skipping have reduced channel activities and could have increased susceptibility for obstructive lung disease. This could be particularly relevant for 5T carriers exposed to additional risk factors for lung disease such as tobacco smoke or familial predisposition to lung disease. Variations in the genes for mannose-binding lectin and α_1_-antitrypsin have been studied as modifiers of cystic fibrosis lung disease [35-37] and could also potentially influence risk of lung disease in 5T heterozygotes. Allele frequencies in whites are approximately 5% for the 5T allele, 84% for 7T, and 11% for 9T [25,26].

We hypothesised that carriers of the 5T allele have increased annual lung function decline and increased risk for asthma or COPD. To test this hypothesis, we genotyped 9131 individuals from the adult Danish population for the 5T, 7T, and 9T alleles in the *CFTR *gene. We combined polythymidine and F508del genotypes [1], and examined associations between 11 different genotype combinations, and annual FEV_1 _decline and risk of asthma or COPD. We also examined whether other common risk factors for lung disease or variations in the genes for mannose-binding lectin and α_1_-antitrypsin significantly add to risk of lung disease in 5T carriers.

## Methods

Subjects participated in the 1976–78, 1981–83, and/or 1991–94 examination of the Copenhagen City Heart Study, a prospective epidemiological study initiated in 1976–78 [38]. Participants aged 20 years and above were selected randomly after age stratification into 5-year age groups from among residents of Copenhagen. Of the 17180 individuals invited, 10135 participated, 9259 gave blood, and 9131 were genotyped for the polythymidine tract variants of the *cystic fibrosis conductance membrane regulator *(*CFTR*) gene. Details of study procedures and some characteristics of non-responders are described elsewhere [38,39]. More than 99% were Whites of Danish descent. All participants gave written informed consent, and Herlev University Hospital and the ethics committee for Copenhagen and Frederiksberg approved the study (# 100.2039/91).

Participants filled out a self-administered questionnaire, which was validated by the participant and an investigator on the day of attendance. Participants reported on long-term occupational exposure to dust or welding fumes, pulmonary symptoms (dyspnea, wheezing, bringing up phlegm), familial predisposition to asthma (having at least one sibling with asthma), smoking habits (current smoker, ex-smoker, never-smoker), type of smoking and daily tobacco consumption. An estimate of life-time tobacco exposure (in packyears) was calculated as: daily tobacco consumption (g) times duration of smoking (years) divided by 20 (g/pack). If at least once during the study period participants aswered "Yes" to the question "Do you suffer from asthma?", we recorded they had self-reported asthma. Medication for asthma / bronchitis was "Yes" to the question "Do you daily take medication for asthma / bronchitis?" Additional information on hospitalizations due to asthma (ICD8: 493; ICD10: J45–46) and COPD (ICD8: 491–492; ICD10: J41–44) was drawn from the Danish National Hospital Discharge Register from May 1st 1976 through December 31st 2000. We confirmed in the Danish National Hospital Discharge Register covering all hospital discharges in Denmark, that no participants in the sample were ever hospitalized for cystic fibrosis.

Forced expiratory volume in one second (FEV_1_) and forced vital capacity (FVC) were measured with an electronic spirometer (model N403, Monaghan, Littleton, Colo.) at the 1976–78 and 1981–83 examinations and with a dry wedge spirometer (Vitalograph, Maidenhead, UK) at the 1991–94 examination. At each examination, three sets of values were obtained, and as a criterion for correct performance of the procedure, at least two measurements of FEV_1 _and FVC differing by less than 5% had to be produced. The highest set of FEV_1 _and FVC were used in the analyses as percentage of predicted value using internally derived reference values based on a subsample of healthy never smokers [40]. Annual decline in FEV_1 _(ml/year) was calculated as FEV_1 _(ml) obtained at the latest measurement minus the FEV_1 _value obtained at the first measurement, times 365.25 divided by the number of days between the two measurements (in years^-1^). Spirometry defined COPD was FEV_1_<80% predicted and FEV_1_/FVC<0.7, excluding self-reported asthma [41].

We amplified the polythymidine tract variants in intron-8 by nested polymerase chain reaction using the primerpairs: 5'-TAATGGATCATGGGCCATGT-3'and 5'-ACAGTGTTGAATGTGGTGCA-3' (first step reaction), and 5'-CCGCCGCTGTGTGTGTGTGTGTGTTTTT-3' and 5'GCTTTCTCAAATAATTCCCC-HEX-3' (second step reaction) (mismatch underlined) [8]. Products of 52 bp (5T allele), 53 bp (6T allele), 54 bp (7T allele), and 56 bp (9T allele) were seperated by capillary electrophoresis on an ABI 310 sequenator. Tamra 350 marker was added to samples before analysis, and each analysis ran dummy standard, water control, and positive controls. The F508del allele in the *CFTR *gene [1], S and Z alleles in the *Serine Protease Inhibitor-A1 *gene [42], and B, C, and D alleles in the *Mannose-Binding Lectin-2 *gene [43] were identified using polymerase chain reaction followed by restriction enzyme digestion as described. Diagnoses of polythymidine alleles in 5T/F508del genotypes, 5T/5T, 6T/7T, and 69 randomly selected 5T/9T, 7T/9T, 7T/7T, 5T/7T, 9T/9T genotypes were confirmed by sequencing. All 7T/7T F508del genotypes were re-analyzed to confirm their diagnosis, using sequencing (7T/7T) and RFLP-PCR (F508del). The number of TG repeats adjacent to the 5T allele in 5T/F508del and 5T/5T genotypes were determined by sequencing. For each polythymidine allele, expected exon-9 skipping was half the middle value of the ranges of skipping observed in homozygotes [32]; expected exon-9 skipping was not estimated in individuals with F508del heterozygosity.

Linkage disequilibrium between the 9T and F508del alleles was tested by the linkage utility program "EH" , which estimates allele and haplotype frequencies with and without allelic association. The linkage disequilibrium coefficient D was calculated as D = P_22 _- p_2_q_2_, where P_22 _is the observed frequency of the 9T/F508del haplotype, p_2 _is the frequency of the F508del allele in the general population and q_2 _is the population frequency of the 9T allele. The degree of linkage disequilibrium was expressed as D' = D/D_max _× 100%.

Statistical analysis was performed with SPSS; for power calculations, NCSS-PASS and StatMate were used. P < 0.05 on a two-sided test was considered significant. Pearson's χ^2^-test or analysis of variance (ANOVA) was used for overall comparisons between several genotypes; Pearson's or Fisher's Exact χ^2^-test were used for post-hoc two-genotype comparisons. The most common genotype combination in the population, 7T homozygosity without F508del, was used as reference group for statistical comparisons. We evaluated asthma and COPD prevalences between genotypes using unadjusted and adjusted logistic regression with Wald's test as a measure of significance; the adjusted model included gender, age at study entry (deciles), and packyears at study entry (never smokers and deciles). We evaluated asthma and COPD incidences between genotypes using the log-rank test [42-44]. Unadjusted and adjusted Cox regression with forced entry examined time to disease by using hazard ratios (relative risks) and 95% confidence intervals; the adjusted model included gender, age at study entry (deciles), tobacco use during follow-up (never smokers and deciles), and FEV_1 _% predicted at study entry (deciles). We tested possible interactions between the 5T/7T genotype and smoking habits, long-term occupational exposure to dust or welding fumes, familial predisposition to asthma, α_1_-antitrypsin MS genotype, α_1_-antitrypsin MZ genotype, or mannose-binding lectin deficiency in predicting FEV_1 _at study entry in ANCOVA models.

## Results

Characteristics of participants are given in Table 1; genotypes are ordered according to predicted increased skipping of exon-9 of the *cystic fibrosis transmembrane conductance regulator *gene, stratified for presence or absence of F508del heterozygosity. Among the 9,131 participants selected randomly from the Danish general population, 352 (3.9%) were 5T heterozygotes and 249 (2.7%) were F508del heterozygotes. Expected numbers of 5T and F508del heterozygotes according to the Hardy Weinberg equilibrium were 349 and 246, respectively. Allele frequencies did not differ from those predicted by the Hardy Weinberg equilibrium (χ^2^-test for 7T allele: P = 0.84; 9T allele: P = 0.60; 6T allele: P = 0.98; 5T allele: P = 0.42; F508del allele: P = 0.19). The novel intron-8 polythymidine tract variant, the 6T allele [45], was identified in four individuals. The 9T and F508del alleles were in linkage disequilibrium with a degree of linkage of 98% (χ^2^-test: P < 0.001).

**Table 1:** Characteristics of subjects by intron-8 polythymidine tract and F508del genotype

Polythymidine	*9T/9T*	*7T/9T*	*7T/7T*	*6T/7T*	*5T/9T*	*5T/7T*	*5T/5T*	*9T/9T*	*7T/9T*	*7T/7T*	*5T/9T*	
Expected exon-9 skipping, %	7	13	18	≥18	43	48	78	-	-	-	-	
F508del heterozygosity								yes	yes	yes	yes	P-value
Women / Men	44 / 39	841 / 699	3,818 / 3,087	2 / 2	22 / 18	171 / 137	1 / 1	13 / 10	127 / 90	4 / 1	2 / 2	0.99
Genotype frequency, %	0.9	16.9	75.6	0.0	0.4	3.4	0.0	0.3	2.4	0.1	0.0	
Smoking before study entry, packyears*	16 ± 2.1	16 ± 0.5	15 ± 0.2	13 ± 10	18 ± 3.0	14 ± 1.1	8.4 ± 12	13 ± 4.0	14 ± 1.3	18 ± 10	14 ± 10	0.81
Age at study entry, years	46 ± 1.4	47 ± 0.3	47 ± 0.2	46 ± 6.3	47 ± 2.0	46 ± 0.7	39 ± 8.9	48 ± 2.6	48 ± 0.9	41 ± 5.6	46 ± 6.3	0.63
FEV_1 _at study entry, %pred.	87 ± 1.9	90 ± 0.4	90 ± 0.2	83 ± 8.8	96 ± 2.8	90 ± 1.0	84 ± 12	94 ± 3.7	89 ± 1.2	84 ± 7.9	101 ± 8.8	0.24
Smoking during follow-up, g/day^†^	9.0 ± 1.1	8.8 ± 0.3	8.9 ± 0.1	11 ± 5.0	8.1 ± 1.6	7.5 ± 0.6	6.3 ± 7.1	7.9 ± 2.1	7.1 ± 0.7	8.0 ± 4.5	8.0 ± 5.0	0.24
Follow-up, years	23 ± 0.14	23 ± 0.03	23 ± 0.02	23 ± 0.66	23 ± 0.21	23 ± 0.08	24 ± 0.93	23 ± 0.27	23 ± 0.09	24 ± 0.59	24 ± 0.66	0.97

Values are number of individuals, percentages, or mean ± SD. P-values by Pearson's χ^2 ^test or analysis of variance. *Calculated as daily tobacco use (g/day) × duration of smoking (years) / 20 (g/pack). ^†^The average amount of tobacco used (in g/day) at the different examinations attended.

### Annual decline in FEV_1_

**Figure 1:**
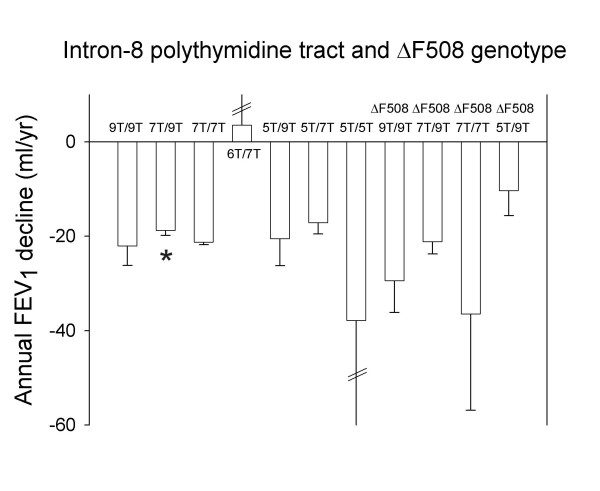
**Annual FEV_1 _decline by intron-8 polythymidine tract and F508del genotype. **Values are mean and SEM. *P = 0.03 compared with 7T homozygotes without F508del.

Annual decline in FEV_1 _did not differ between 5T heterozygotes or homozygotes vs. 7T homozygous controls (Fig. 1). 7T/9T individuals had annual decline in FEV_1 _of 19 ml compared with 21 ml in 7T homozygous controls (t-test: P = 0.03; Fig. 1). None of the other genotype combinations differed from 7T homozygous controls. The analysis had 90% power to detect differences in annual FEV_1 _decline of 14 ml in 9T/9T, 3.8 ml in 7T/9T, 61 ml in 6T/7T, 23 ml in 5T/9T, 8 ml in 5T/7T, 31 ml in 9T/9T F508del, 9 ml in 7T/9T F508del, 72 ml in 7T/7T F508del, and 72 ml in 5T/9T F508del individuals vs. 7T homozygous controls.

### Asthma

Prevalence of self-reported asthma did not differ between 5T heterozygotes or homozygotes vs. 7T homozygous controls (Ps ≥ 0.10; data not depicted). However, self-reported asthma differed between genotypes overall (χ^2^: P = 0.02); eleven percent of 7T/9T individuals with F508del (χ^2^: P = 0.01) and 40% of 7T homozygotes with F508del (χ^2^: P = 0.04) had asthma vs. 6.7% of 7T homozygous controls (data not depicted). None of the other genotype combinations differed from 7T homozygous controls.

**Figure 2:**
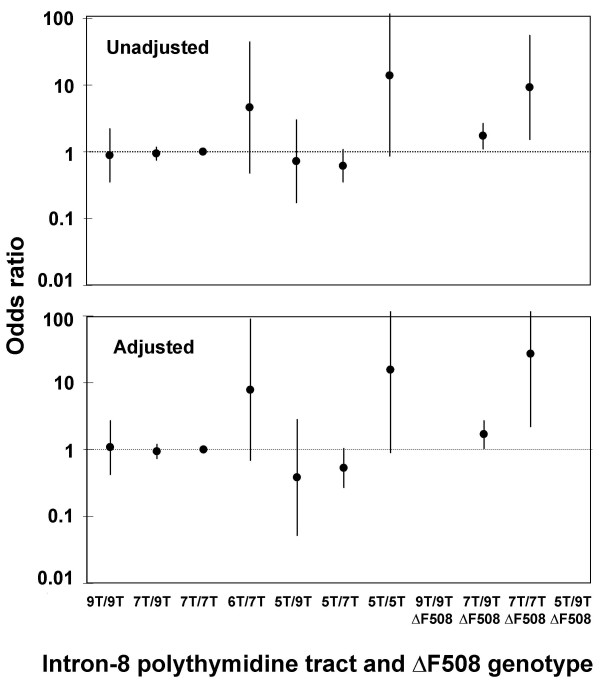
**Odds ratios for self-reported asthma by intron-8 polythymidine tract and F508del genotype. **7T homozygotes without F508del was used as reference group. The adjusted model included gender, age at study entry, and packyears at study entry. Error bars are 95% confidence intervals. Self-reported asthma = "Yes" at least once during the study period to the question "Do you suffer from asthma?".

Unadjusted odds ratios for self-reported asthma were 1.7 (95%CI:1.1–2.7) in 7T/9T individuals with F508del and 9.2 (1.5–55) in 7T homozygotes with F508del vs. 7T homozygous controls (Fig. 2, upper panel). After adjusting for gender, age at study entry, and packyears at study entry, equivalent odds ratios for self-reported asthma were 1.7 (1.0–27) in 7T/9T individuals with F508del and 27 (2.2–327) in 7T homozygotes with F508del (Fig. 2, lower panel). The analysis had 90% power to detect an odds ratio for asthma of 3.0 for 9T/9T, 1.4 for 7T/9T, 23 for 6T/7T, 4.2 for 5T/9T, 1.9 for 5T/7T, 5.8 for 9T/9T F508del, 2.1 for 7T/9T F508del, 18 for 7T/7T F508del, and 23 for 5T/9T F508del individuals vs. 7T homozygous controls.

Incidence of hospitalization from asthma during 24 years follow-up did not differ between 5T heterozygotes or homozygotes versus 7T homozygous controls (Table 2). However, incidence of asthma hospitalization was increased in 7T homozygotes with F508del compared with 7T homozygous controls (Table 2). Unadjusted and after adjusting for gender, age at study entry, tobacco consumption, and FEV_1 _% predicted at study entry, the hazard ratio for asthma hospitalization was 11 (1.5–78) and 6.3 (0.84–47) in 7T homozygotes with F508del vs. 7T homozygous controls. None of the other genotype combinations differed from 7T homozygous controls (Table 2). The analysis had 90% power to detect a hazard ratio for asthma hospitalization of 2.7 for 9T/9T, 1.4 for 7T/9T, 15 for 6T/7T, 3.7 for 5T/9T, 1.8 for 5T/7T, 4.9 for 9T/9T F508del, 2.0 for 7T/9T F508del, 13 for 7T/7T F508del, and 15 for 5T/9T F508del individuals vs. 7T homozygous controls.

**Table 2:** Incidences and hazard ratios for asthma hospitalisation by intron-8 polythymidine tract and F508del genotype during 24 years follow-up

Poly-T	Expected exon-9 skipping, %	F508del heterozygosity	n	Incidence n/10000 person-years	P-value*	Unadjusted HR (95%CI)	Adjusted^† ^HR (95%CI)	90% power^‡ ^HR
*9T/9T*	7		83	9.8	0.83	1.2 (0.28–4.7)	1.1 (0.27–4.4)	2.7
*7T/9T*	13		1540	9.3	0.60	1.1 (0.76–1.6)	1.1 (0.77–1.6)	1.4
*7T/7T*	18		6905	8.4	-	1.0	1.0	-
*6T/7T*	≥18		4	0	0.77	-	-	15
*5T/9T*	43		40	10	0.85	1.2 (0.17–8.6)	1.2 (0.17–8.9)	3.7
*5T/7T*	48		308	5.3	0.35	0.63 (0.23–1.7)	0.53 (0.17–1.7)	1.8
*5T/5T*	78		2	0	0.84	-	-	25
*9T/9T*	-	yes	23	0	0.49	-	-	4.9
*7T/9T*	-	yes	217	11	0.47	1.3 (0.59–3.1)	1.3 (0.55–2.9)	2.0
*7T/7T*	-	yes	5	87	0.003	11 (1.5–78)	6.3 (0.84–47)	13
*5T/9T*	-	yes	4	0	0.77	-	-	15

*P-values are for the comparison with 7T/7T individuals without the F508del deletion by log-rank test. ^†^Cox regression adjusted for gender, age at study entry, tobacco use during follow-up, and FEV_1 _% predicted at study entry. ^‡^90% power to detect a hazard ratio (HR) of asthma at 2-sided P < 0.05. 95%CI = 95% confidence interval. Hospitalizations from asthma (ICD8: 493; ICD10: J45–46) were drawn from the Danish National Discharge Register from 1976 through 2000.

### Chronic obstructive pulmonary disease (COPD)

**Figure 3:**
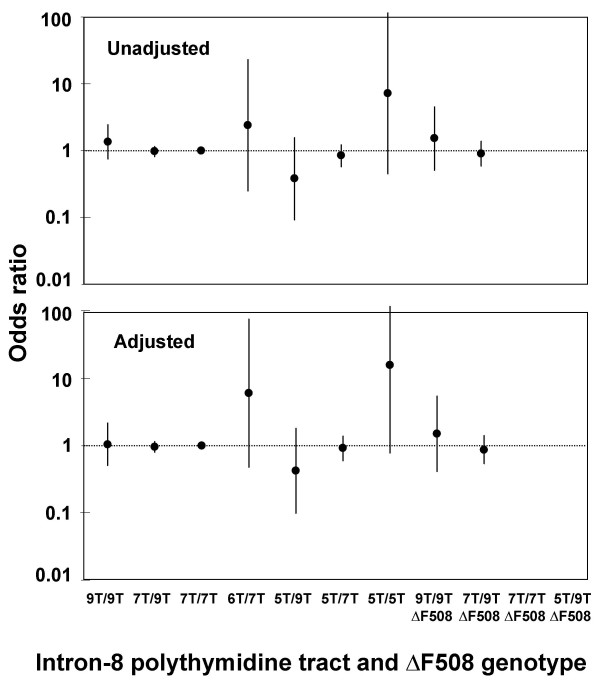
**Odds ratios for spirometry defined COPD by intron-8 polythymidine tract and F508del genotype. **7T homozygotes without F508del was used as reference group. The adjusted model included gender, age at study entry, and packyears at study entry. Error bars are 95% confidence intervals. COPD = FEV_1_<80% predicted and FEV_1_/FVC<0.7, excluding self-reported asthma.

Prevalence of spirometry defined COPD did not differ between 5T heterozygotes or homozygotes vs. 7T homozygous controls (Ps ≥ 0.22) and did not differ between genotypes overall (χ^2^: P = 0.51) (data not depicted). Unadjusted and adjusted odds ratios for spirometry defined COPD did not differ between genotypes (Fig. 3). The analysis had 90% power to detect an odds ratio for COPD of 2.5 for 9T/9T, 1.3 for 7T/9T, 19 for 6T/7T, 3.4 for 5T/9T, 1.7 for 5T/7T, 4.6 for 9T/9T F508del, 1.8 for 7T/9T F508del, 15 for 7T/7T F508del, and 19 for 5T/9T F508del individuals vs. 7T homozygous controls.

Incidence of hospitalization from COPD during 24 years follow-up was reduced in 5T/7T individuals vs. 7T homozygous controls (Table 3). Unadjusted and after adjusting for gender, age at study entry, tobacco consumption and FEV_1 _% predicted at study entry, the hazard ratio for COPD was 0.47 (0.23–0.95) and 0.49 (0.23–1.0) in 5T/7T individuals vs. 7T homozygous controls (Table 3). There was a trend toward increased incidence of COPD hospitalization in 6T/7T individuals; unadjusted and adjusted hazard ratio for COPD hospitalization was 4.9 (0.69–35) and 7.6 (1.0–55) in 6T/7T individuals vs. 7T homozygous controls (Table 3). Other genotypes did not differ in COPD risk from 7T homozygous controls. The analysis had 90% power to detect a hazard ratio for COPD of 2.3 for 9T/9T, 1.3 for 7T/9T, 11 for 6T/7T, 3.0 for 5T/9T, 1.6 for 5T/7T, 3.8 for 9T/9T F508del, 1.7 for 7T/9T F508del, 9.7 for 7T/7T F508del, and 11 for 5T/9T F508del individuals vs. 7T homozygous controls.

**Table 3:** Incidences and hazard ratios for COPD hospitalisation by intron-8 polythymidine tract and F508del genotype during 24 years follow-up

Poly-T	Expected exon-9 skipping, %	F508del heterozygosity	n	Incidence n/10000 person-years	P-value*	Unadjusted HR (95%CI)	Adjusted^† ^HR (95%CI)	90% power^‡ ^HR
*9T/9T*	7		83	40	0.10	1.8 (0.89–3.6)	1.7 (0.85–3.5)	2.3
*7T/9T*	13		1540	21	0.70	0.95 (0.75–1.2)	0.99 (0.78–1.3)	1.3
*7T/7T*	18		6905	22	-	1.0	1.0	-
*6T/7T*	≥18		4	105	0.08	4.9 (0.69–35)	7.6 (1.0–55)	11
*5T/9T*	43		40	21	0.90	0.92 (0.23–3.7)	0.75 (0.19–3.0)	3.0
*5T/7T*	48		308	11	0.03	0.47 (0.23–0.95)	0.49 (0.23–1.0)	1.6
*5T/5T*	78		2	0	0.73	-	-	19
*9T/9T*	-	yes	23	0	0.25	-	-	3.8
*7T/9T*	-	yes	217	25	0.73	1.1 (0.63–1.9)	1.1 (0.62–1.9)	1.7
*7T/7T*	-	yes	5	0	0.59		-	9.7
*5T/9T*	-	yes	4	0	0.63	-	-	11

*P-values are for the comparison with 7T/7T individuals without the F508del deletion by log-rank test. ^†^Cox regression adjusted for gender, age at study entry, tobacco use during follow-up, and FEV_1 _% predicted at study entry. ^‡^90% power to detect a hazard ratio (HR) of COPD at 2-sided P < 0.05. 95%CI = 95% confidence interval. Hospitalizations from COPD (ICD8: 491–492; ICD10: J41–44) were drawn from the Danish National Discharge Register from 1976 through 2000.

### 5T homozygotes and 5T/F508del compound heterozygotes

One of two 5T homozygous smokers reported having asthma and took daily medication for respiratory disease (Table 4). The other homozygous individual showed evidence of airway obstruction with reversibility and was referred for further examination and treatment of asthma. None of four 5T/F508del compound heterozygotes had clinical signs of severe pulmonary disease (Table 4).

**Table 4:** Pulmonary status of 5T homozygotes and 5T/F508del compound heterozygotes sampled from the general population

Poly-T*	F508del heterozygosity	Age	Gender	Smoking status	FEV_1_		Self-reported asthma^‡^	Medication for asthma / bronchitis^¶^	Hospitalization		Often bothered by		
									
		years			%predicted	reversibility^†^			asthma**	COPD**	dyspnoea	wheezing	phlegm
*TG12-5T/TG12-5T*		32	M	current smoker	92	-	yes	yes	no	no	yes	yes	no
*TG11-5T/TG11-5T*		62	F	current smoker	67	30%	no	no	no	no	no	no	no
*TG11-5T*	yes	33	F	current smoker	115	-	no	no	no	no	no	no	no
*TG11-5T*	yes	62	M	never smoker	121	-	no	no	no	no	no	no	no
*TG12-5T*	yes	65	F	ex-smoker	79	-	no	no	no	no	no	no	no
*TG11-5T*	yes	70	M	current smoker	128	-	no	no	no	no	no	no	no

*Number of TG repeats adjacent to the polythymidine tract included. ^†^FEV_1 _30 minutes after inhalation of 0.5 mg terbutaline minus FEV_1 _at 0 minutes divided by FEV_1 _at 0 minutes times 100%; only individuals with FEV_1_/FVC<0.7 were tested for FEV_1 _reversibility. ^‡^"Yes" to "Do you suffer from asthma?" ^¶^"Yes" to "Do you daily take medication for asthma / bronchitis?" **Hospitalizations from asthma (ICD8: 493; ICD10: J45–46) and COPD (ICD8: 491–492; ICD10: J41–J44) were drawn from the Danish National Discharge Register from 1976 through 2000.

### Context-dependent associations for 5T/7T genotype

There was no interaction between 5T/7T genotype and smoking status (P = 0.78), occupational exposure to dust or welding fumes (P = 0.10), familial asthma (P = 0.37), α_1_-antitrypsin MS genotype (P = 0.64), α_1_-antitrypsin MZ genotype (P = 0.47), or mannose-binding lectin deficiency (P = 0.73) in predicting FEV_1 _% predicted at study entry.

## Discussion

This study shows that polythymidine 5T heterozygosity is not associated with increased annual decline in FEV_1 _or risk of asthma or COPD in the adult Caucasian population; these results are independent of age, gender, tobacco smoking, and other potential confounders. Interestingly, however, both 5T homozygotes showed evidence of asthma. Furthermore, our results support that F508del heterozygosity is associated with increased asthma risk independently of the 5T allele.

Because 1 in 26 carries a 5T allele in this population, it is indeed important that 5T heterozygosity does not increase risk of obstructive lung disease in the population at-large. It appears that the 5T allele causes lung disease only in very rare circumstances [9-14], leaving the average heterozygous individual unaffected by obstructive lung disease. Previous results suggest that penetrance of pulmonary manifestations in 5T carriers might depend on the length of an adjacent TG repeat [46,47]. This could be particularly relevant for 5T homozygotes and compound heterozygotes. In 5T heterozygotes, however, longer TG repeats seem less likely to affect risk of pulmonary disease. This is because 5T heterozygosity was not associated with risk of lung disease in this study although predicted TG12 and TG13 allele frequency in 5T carriers in our population was 31% [47]. Other additional genetic variations have also been shown to influence exon-9 skipping in 5T carriers, but to a lesser degree than the TG repeat.

Because all 5T/F508del compound heterozygotes were free from severe pulmonary disease, the 5T allele did not appear to explain our previous results [1,6] suggesting that F508del heterozygosity may be overrepresented among asthmatics. A few recent studies also support this observation [2,19,48], while others have found no [20,21,49] or negative associations [50]. In the present analyses, 7T/9T and 7T/7T individuals with F508del heterozygosity had higher prevalences of self-reported asthma, and 7T/7T individuals with F508del heterozygosity also had higher incidence of hospitalization from asthma. F508del heterozygosity was only associated with increased asthma risk in individuals without the 5T allele, indicating that our previous observations are independent of influence from this allele. In addition, both 5T homozygotes showed evidence of asthma supporting the hypothesis that CFTR variations may be associated with asthma [2,19].

To identify factors in the population that significantly add to risk of lung disease in 5T heterozygotes, we tested for interactions between 5T/7T genotype and potential risk factors for lung disease, but found no significant interactions. Garred [35] and coworkers found a worse prognosis in cystic fibrosis patients with MBL deficiency. We were not able to extend this finding, since lung function in 5T or F508del heterozygotes was not reduced by MBL deficiency. Previous studies by Mahadeva [36] and Frangolias [37] showed that pulmonary disease severity in cystic fibrosis patients were unaffected by α_1_-antitrypsin S and Z alleles. In line with this, we also observed no increased risk for pulmonary dysfunction in 5T carriers with α_1_-antitrypsin MS or MZ genotypes.

In the present study, bias caused by investigators' knowledge of disease or risk-factor status seems unlikely, because we selected from a general population and genotyped our sample without knowledge of disease status or lung function test results. Selection bias is possible if severe lung disease in some individuals with 5T genotypes prevented them from participating in our study; however, expected and observed numbers of these genotypes according to the Hardy-Weinberg equilibrium were similar. The 2.7% frequency of F508del heterozygosity found in this study is in accordance with the 2.9% frequency of F508del heterozygosity observed in another previous study of the Danish population [51]. Annual decline in FEV_1 _was reduced in 7T/9T individuals and incidence of COPD hospitalization was reduced in 5T/7T individuals. If correction for multiple comparisons was performed, these significant findings become nonsignificant. Therefore, and because reduced COPD risk in 5T/7T individuals is less biologically plausible, the findings are likely due to chance alone rather than representing real phenomena. Misclassification of genotypes is unlikely, because diagnoses were confirmed by sequencing a subsample of different poly-T variants.

## Conclusion

Polythymidine 5T heterozygosity was not associated with increased annual decline in FEV_1 _or risk of asthma or COPD in adults in this population-based study; however, both 5T homozygotes showed evidence of asthma. Furthermore, our results also support that F508del heterozygosity may be associated with increased asthma risk independently of the 5T allele.

## Competing interests

The author(s) declare that they have no competing interests.

## Authors' contributions

Morten Dahl, Anne Tybjærg-Hansen, and Børge G. Nordestgaard carried out the genotyping and statistical analysis. Peter Lange helped collect the data and was involved in the statistical analysis. All investigators participated in designing the study and in writing the paper, and all authors read and approved the final version of the manuscript.
